# Colorectal cancer awareness and acceptance of fecal immunochemical test screening in Qassim, Saudi Arabia

**DOI:** 10.3389/fpubh.2026.1710204

**Published:** 2026-01-21

**Authors:** Bader Alshamsan, Tasneem Alajlan, Reema Alsweed, Unaib Rabbani

**Affiliations:** 1 Department of Medicine, College of Medicine, Qassim University, Qassim, Saudi Arabia; 2 Health and Curative Programs Department, Public Health and Community Health Administration, Qassim Health Cluster, Buraidah, Saudi Arabia; 3 Family Medicine Academy, Qassim Health Cluster, Buraidah, Saudi Arabia

**Keywords:** cancer awareness, colorectal cancer, fecal immunochemical test, health literacy, Saudi Arabia, screening adherence

## Abstract

**Background:**

Saudi Arabia recently launched a national colorectal cancer (CRC) screening program. Successful control of CRC depends not only on public acceptance but also on sustained adherence and adequate knowledge of CRC symptoms and risk factors. This study evaluated the acceptance, adherence, and knowledge of CRC in the Qassim region, and explored the barriers and facilitators of fecal immunochemical test (FIT) uptake.

**Methods:**

A cross-sectional survey of 2,050 average-risk adults aged 45–75 years was conducted at Qassim between December 2024 and February 2025. A structured questionnaire was used to collect socio-demographics, screening history, perceived barriers and facilitators, and knowledge of CRC symptoms and risk factors, utilizing a validated Cancer Awareness Measure (CAM). Logistic regression was used to identify predictors of FIT uptake.

**Results:**

Acceptance of FIT was high (88.3%), but adherence to annual testing was low (43.1%). Knowledge of CRC was poor (mean CAM score 8.2/22), and only 15.2% showed good knowledge. In the multivariable analysis, reduced uptake was independently associated with poorer self-perceived health (OR = 0.19, 95% CI 0.05–0.78) and low CRC knowledge (poor: OR = 0.34, 95% CI 0.16–0.73; intermediate: OR = 0.21, 95% CI 0.09–0.46), whereas more frequent physician visits (2–5/year: OR = 1.57, 95% CI 1.07–2.31; >5/year: OR = 6.07, 95% CI 1.84–19.99), prior screening (OR = 2.02, 95% CI 1.30–3.14) and previous FIT (OR = 6.50, 95% CI 3.61–11.69) predicted higher uptake. Misconceptions, fear, and logistical issues limited participation, while mailed kits, electronic results, and physician counseling encouraged uptake.

**Conclusion:**

In Qassim, FIT screening was widely accepted but poorly maintained, and CRC knowledge was low. National control of CRC will require accessible, patient-centered services and stronger public knowledge of symptoms and risk factors to support prevention, regular screening, and timely help-seeking.

## 1 Introduction

Colorectal cancer (CRC) is the third most common malignancy worldwide and the fifth leading cause of cancer-related death (1, 2). Globally, CRC incidence varies widely, with rates stabilizing in high-income countries but continuing to rise in low- and middle-income countries owing to lifestyle changes such as obesity, inactivity, and dietary patterns (3–5). In Saudi Arabia, CRC represents a major and rapidly escalating public health challenge. Data from the Saudi Cancer Registry and GLOBOCAN 2022 indicate that CRC is the most frequently diagnosed cancer in men and a leading cause of cancer-related mortality in both sexes (6, 7). Between 2002 and 2016, the age-standardized incidence rate increased by an average annual percentage change of 6.1% nationwide, with an even steeper increase observed in the Qassim region (7.8%) during the same period (8, 9). In 2023, age-standardized incidence rates reached 24.1 per 100,000 in men and 19.9 per 100,000 in women, exceeding rates reported in the Gulf Cooperation Council and neighboring countries, but remaining lower than those observed in many Western nations (10). Notably, this rapid increase places Saudi Arabia among the countries with the fastest-growing CRC burden worldwide (11, 12). This trend is largely attributed to population aging, urbanization, dietary changes, physical inactivity, and rising obesity prevalence (11–14). Established risk factors include alcohol, tobacco, red and processed meat, and excess body fat, whereas protective factors include fiber, whole grains, dairy, calcium, and regular physical activity (5).

Colonoscopy remains the gold standard for CRC screening; however, it is costly and resource-intensive, particularly in low-resource settings (15). The fecal immunochemical test (FIT) has therefore emerged as a cost-effective and acceptable alternative, with substantial evidence supporting its effectiveness and population uptake (16–18). The awareness of CRC symptoms (e.g., rectal bleeding, altered bowel habits, unexplained anemia, or weight loss) and recognition of modifiable risk factors are critical, since greater knowledge is consistently linked to higher screening uptake and earlier help-seeking (19–25).

To address the growing burden of CRC, the Saudi Ministry of Health launched an organized, population-based CRC screening program in 2017 that uses the FIT as the primary screening modality (26, 27). The program targets average-risk adults aged 45–75 years and offers annual FIT screening, free of charge, through public primary healthcare centers, with referral for diagnostic colonoscopy following a positive test. Nevertheless, effective CRC control depends not only on access to screening but also on public acceptance, sustained adherence, symptom recognition, and awareness of modifiable risk factors. Both prevention and early detection rely on these elements; however, regional data quantifying these factors remain limited.

Therefore, this study aimed to (i) assess the willingness of average-risk adults in the Qassim region to undergo FIT screening and identify key barriers and facilitators, and (ii) evaluate the knowledge and awareness of CRC symptoms and risk factors. These findings will inform strategies to strengthen prevention, promote early detection, and improve the long-term effectiveness of national CRC control efforts.

## 2 Methods

### 2.1 Study design and setting

This cross-sectional study was conducted among adults aged 45–75 years attending primary healthcare (PHC) centers in the Qassim region, Saudi Arabia, between December 1, 2024, and February 28, 2025. Ethical approval was obtained from the Regional Research Ethics Committee, Health and Curative Programs Department, Public Health and Community Health Administration, and Qassim Health Cluster (approval no. 607/46/827); approved on 29 July 2024. Written informed consent was obtained from all participants. This study was designed and reported in accordance with the STROBE (Strengthening the Reporting of Observational Studies in Epidemiology) guidelines for cross-sectional studies (28).

The Qassim region included 149 PHC centers in the public sector that formed our sampling frame. An ongoing national CRC screening program provides a non-invasive, free-of-charge FIT. Individuals with positive results are referred for colonoscopy and further management. Eligible participants were adults aged 45–75 years who met the national criteria for average-risk CRC screening, had not undergone FIT in the preceding 12 months, and were able to provide informed consent and independently complete the survey. Participants were excluded if they were classified as high-risk (defined as CRC symptoms, inflammatory bowel disease, personal or family history of CRC or advanced adenomas, hereditary CRC syndromes, prior abdominal or pelvic radiation, or serrated polyposis syndrome). Participants were also excluded if they had undergone CRC screening within recommended intervals. These included completion of FIT within the past 12 months, colonoscopy within the previous 10 years, sigmoidoscopy within 5 years, stool DNA testing within 3 years, or CT colonography within 5 years.

The sample size was estimated using the OpenEpi software. Assumptions were informed by regional Ministry of Health CRC screening data from January to June 2024, which indicated an approximate refusal rate of 29% at the 95% confidence level and a 2% margin of error, yielding a minimum sample size of 1,974. To account for an anticipated response rate of 50%, 3,948 individuals were targeted, drawn from an estimated 143,871 adults aged 45–75 years residing in the Qassim region. Participants were recruited using consecutive sampling from all public primary healthcare centers across the Qassim region during routine clinical visits. This pragmatic, non-random approach was chosen to reflect the real-world implementation of the national screening program. All eligible individuals encountered during the study period were approached face-to-face and invited to participate until the target sample size was achieved.

### 2.2 Survey instrument and data collection

Data were collected using a structured, face-to-face interviewer-administered questionnaire adapted from the validated Bowel/Colorectal Cancer Awareness Measure (CAM), developed by the University College London and Cancer Research UK (29).

The questionnaire was administered in Arabic, the participants’ native language. The original English version underwent forward–backward translation by bilingual experts, content review by a multidisciplinary panel, and piloting with 30 adults to ensure clarity, cultural appropriateness and feasibility.

The instrument comprised six parts: Part A (Eligibility screen; 4 items); Part B (sociodemographic and clinical characteristics; 18 items), including age, sex, marital status, education, employment, BMI, smoking, residence (central [main cities] vs. peripheral areas), physical activity, participation in other screening programs, prior FIT uptake, and awareness of the local CRC screening program; Part C (Knowledge of FIT and CRC screening in Qassim; 4 items) covering awareness of the standard test, availability of the free program, recommended starting age, and test interval; Part D (Knowledge of CRC symptoms [10 items] and risk factors [12 items] using CAM) from which we derived the Symptom Knowledge Score (CAM-S), Risk Factor Knowledge Score (CAM-RF), and Total Knowledge Score (CAM-Total; 22 items); Part E (perceived barriers to FIT; 10 items); and Part F (facilitators of screening uptake; 7 items). Appendix 1 provides the English version of the questionnaire. Knowledge scores were categorized as poor, intermediate, or good using predefined thresholds: CAM-S (0–3, 4–7, 8–10), CAM-RF (0–4, 5–8, and 9–12), and CAM-Total (0–9, 10–17, and 18–22).

### 2.3 Statistical analysis

All analyses were performed using SPSS version 30 software (IBM Corp., Armonk, NY, United States). Descriptive statistics (frequencies, percentages, means, and standard deviations) were used to summarize participant characteristics. Internal consistency of the CRC Knowledge Scale and subscales was assessed using Cronbach’s *α*, which showed excellent reliability (α = 0.935 total CAM; *α* = 0.878 CAM-S; *α* = 0.901 CAM-RF). The item–total correlation confirmed that all items contributed meaningfully; the deletion of any item did not improve reliability, supporting the retention of the full scale. Associations between willingness to participate in FIT screening and sociodemographic, clinical, and knowledge variables were examined using the chi-square tests. Variables significant in the univariate analysis were entered into a multivariable logistic regression model to identify independent predictors of FIT uptake. Crude and adjusted odds ratios (ORs) with 95% confidence intervals (CIs) are reported. Statistical significance was set at a two-sided *p*-value <0.05.

## 3 Results

### 3.1 Participant characteristics

**Figure 1:**
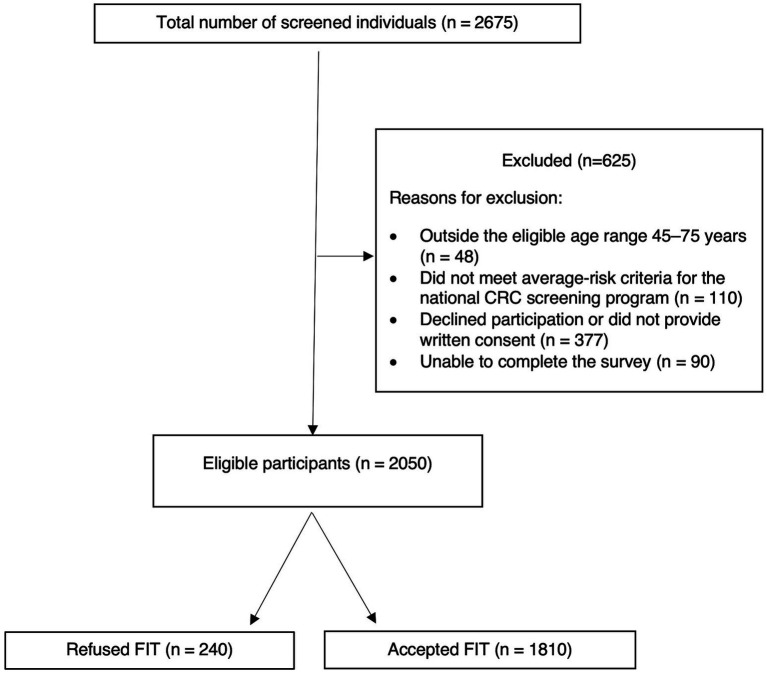
Flow diagram of study participants. Of the 2,675 individuals screened, 2,050 were included in the final analysis. Among these, 1,810 (88.3%) accepted fecal immunochemical test (FIT) screening and 240 (11.7%) declined participation.

Of the 2,675 individuals initially screened, 2,050 met the eligibility criteria and were included in the final analysis. Among them, 1,810 (88.3%) accepted the FIT test, whereas 240 (11.7%) refused to participate (Figure 1). The mean age of the participants was 56.4 years (SD ± 7.6), and 51.6% of the participants were female. The majority were married (92.3%), residing in peripheral areas (59.0%), or were unemployed (46.0%). More than half (55.0%) had completed high school or lower, and 76.9% were overweight or obese (median BMI = 27.6 kg/m^2^). Most participants reported good perceived health (72.8%), were nonsmokers (89.5%), and nearly half (48.9%) reported 2–5 physician visits in the past year. Regarding screening history, 79.0% had previously undergone at least one type of health screening, most commonly for diabetes (69.8%), and hypertension (61.1%). Breast cancer screening was reported by 34.6% of women and osteoporosis screening by 12.6% of participants. Among those with prior FIT screening who responded to the questions on test timing and regularity (*n* = 662), the majority (83.3%) had their last test in the previous year, although only 43.1% reported regular annual FIT uptake. The detailed sociodemographic and clinical characteristics are shown in Table 1.

**Table 1:** Sociodemographic and health characteristics of study participants (n = 2,050).

Characteristic	Category	*N* (%)
Age group	45–54	461 (22.5)
	55–64	1,199 (58.5)
	65–74	388 (18.9)
Sex	Male	993 (48.4)
	Female	1,057 (51.6)
Marital status	Single	30 (1.6)
	Married	1757 (92.3)
	Widowed/Separated	116 (6.1)
Education level	Illiterate	477 (27.2)
	High school or below	963 (55.0)
	University/Postgraduate	311 (17.8)
Employment status	Employed	533 (31.5)
	Self-employed	77 (4.6)
	Retired	304 (18.0)
	Unemployed	778 (46.0)
Residence	Main cities	756 (41.0)
	Peripheral	1,089 (59.0)
Smoking status	Smoker	64 (3.7)
	Ex-smoker	117 (6.8)
	Non-smoker	1,541 (89.5)
Physical activity	Active	450 (24.8)
	Moderate	708 (39.0)
	Sedentary	656 (36.2)
BMI category	Obese	630 (31.1)
	Overweight	927 (45.8)
	Normal	466 (23.0)
Perceived health	Good	1,368 (72.8)
	Moderate	490 (26.1)
	Poor	21 (1.1)
Physician visits (last 12 months)	0–1 time	897 (43.8)
	2–5 times	1,002 (48.9)
	>5 times	151 (7.4)
Any prior screening	Yes	1,619 (79.0)
	No	431 (21.0)
HTN screening	Yes	1,253 (61.1)
	No	797 (38.9)
DM screening	Yes	1,431 (69.8)
	No	619 (30.2)
BC screening	Yes	366 (34.6)
	No	691 (65.4)
Osteoporosis screening	Yes	259 (12.6)
	No	1791 (87.4)
FIT screening	Yes	676 (33.0)
	No	1,374 (67.0)

BMI, Body mass index; DM, Diabetes mellitus; HTN, Hypertension; BC, Breast cancer; FIT, Fecal immunochemical test.

### 3.2 Awareness of colorectal cancer screening in the Qassim region

Awareness of CRC screening programs varies widely among participants. While 52.9% correctly identified FIT as the standard screening method, 40.2% were unsure, and 6.9% believed that FIT was not the standard test. A greater proportion (63.0%) were aware that FIT was offered free of charge through the national program, although 32.5% did not know this, and 4.5% believed it was not free. Knowledge of the recommended starting age for screening was accurate among 56.8% of the respondents, who identified 45 years as the correct starting point, while 24.2% did not know, and the remainder selected older age thresholds. Awareness of the screening interval was limited. Only 8.5% correctly indicated that the FIT should be performed annually. In contrast, 41.7% believed that it was needed only once in their lifetime, and 37.4% did not know the appropriate frequency. Despite meeting the eligibility criteria, only 4.3% of the participants believed that they were at a higher risk for CRC, and more than half (56.9%) were unsure of their personal risk.

### 3.3 Colorectal cancer knowledge: symptoms and risk factors

Participants’ knowledge of CRC symptoms and risk factors was generally limited. The mean total CRC knowledge score was 8.2 out of 22 (SD = 6.6), with a median of 6 and an interquartile range (IQR) of 2–13. Based on predefined categories, 63.0% of the participants had poor overall knowledge, 21.9% had intermediate knowledge, and 15.2% had good knowledge. The mean symptom knowledge score was 3.9 out of 10 (SD = 3.1). Poor knowledge of symptoms was observed in 54.0% of participants, while 25.7% had intermediate knowledge, and 20.3% had good knowledge. The mean risk factor knowledge score was 4.3 out of 12 (SD = 3.9). Poor knowledge of risk factors was reported by 58.6%, intermediate knowledge by 22.6%, and good knowledge by 18.8%. The frequency of correct answers for the CAM total (22 items) is presented in Table 2.

**Table 2:** Colorectal cancer awareness measure (CAM): symptom (CAM-S) and risk factor (CAM-RF), *n* = 2050.

CAM-total items	Correct answer *n* (%)
Symptoms (CAM-S)	
Seek help promptly	1,663 (81.1)
Blood in stool	1,050 (51.2)
Abdominal lump	955 (46.6)
Weight loss	839 (40.9)
Abdominal pain	697 (34.0)
Bowel habit change	674 (32.9)
Rectal pain	644 (31.4)
Anemia/fatigue	639 (31.2)
Incomplete emptying	550 (26.8)
Confidently noticing symptoms	245 (12.0)
Risk factors (CAM-RF)	
Smoking	1,154 (56.3)
Alcohol	808 (39.4)
Low fruit/vegetable intake	801 (39.1)
Overweight (BMI ≥ 25)	758 (37.0)
Family history of CRC	752 (36.7)
Low fiber diet	731 (35.7)
Red/processed meat	726 (35.4)
IBD (UC, Crohn’s)	618 (30.1)
Diabetes	611 (29.8)
Older age (>70 years)	503 (24.5)
Low physical activity	501 (24.4)
Perceived age most at risk	245 (12.0)

CAM, Cancer awareness measure; CAM-S, Symptom knowledge items; CAM-RF, Risk factor knowledge items; CAM-Total, Total knowledge items; IBD, Inflammatory bowel disease; UC, Ulcerative colitis.

Education level was significantly associated with knowledge (*p* = 0.002), with good knowledge reported by 19.0% of those with university or postgraduate education, compared to 11.1% of illiterate participants and 17.8% of those with high school or below. Perceived health status was also significant (p = 0.002), with only 4.8% of participants with poor health reporting good knowledge, compared to 12.0% with moderate health and 18.3% with good health. Perceived risk of CRC showed the strongest association (*p* < 0.001): participants who considered themselves at higher risk reported good knowledge most frequently (39.8%), compared to 20.7% of those responding “no” and 9.5% of those responding “I do not know”.

### 3.4 Refusal rate and barriers to participation in FIT-based colorectal cancer screening

Of the 2,050 participants, 240 (11.7%) declined to undergo FIT screening. The most frequent barriers were the belief that screening was unnecessary in the absence of symptoms (49.2%) or a family history of cancer (43.3%). Other reasons included fear of a positive result (36.3%), embarrassment (29.6%), inconvenience (31.3%), lack of time (29.2%), hesitancy toward colonoscopy follow-up (31.7%), and doubts regarding FIT effectiveness (14.2%).

**Figure 2:**
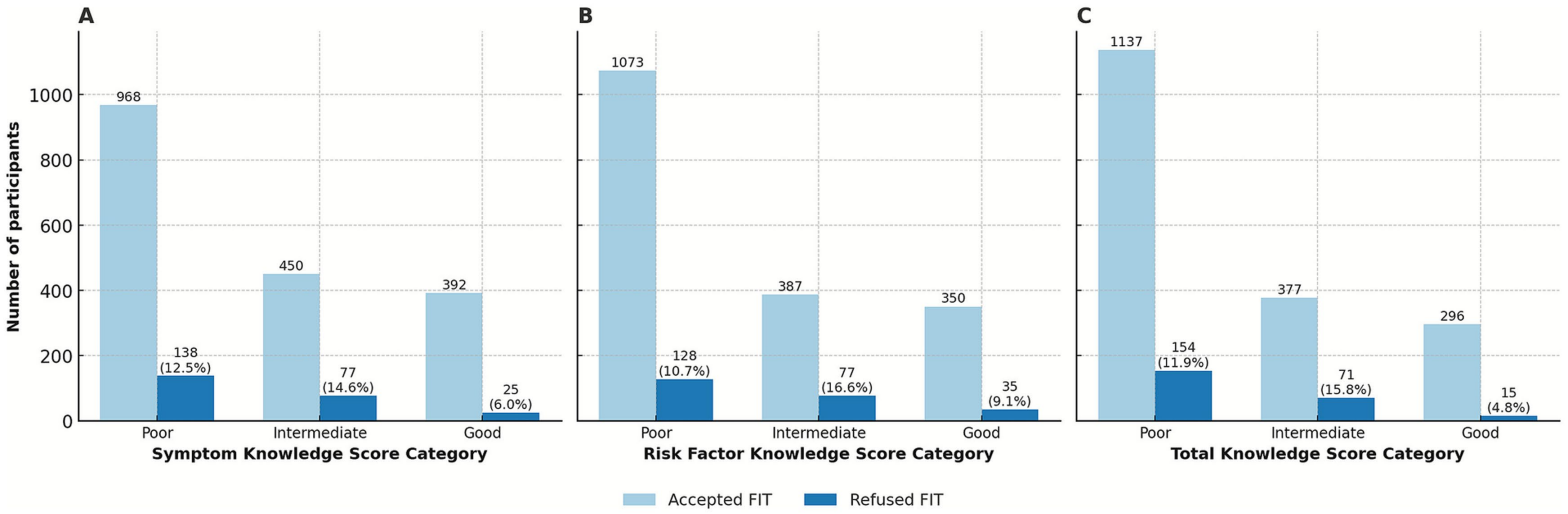
Uptake of FIT screening stratified by colorectal cancer awareness scores. Acceptance and refusal are shown according to **(A)** Symptom Knowledge Score (CAM-S), **(B)** Risk Factor Knowledge Score (CAM-RF), and **(C)** Total Knowledge Score (CAM-Total). FIT, fecal immunochemical test; CAM, Cancer Awareness Measure.

In the univariate analysis, higher FIT participation was observed among women, unemployed individuals, participants with more frequent physician visits, those with a history of other health screenings, and individuals who had previously undergone FIT screening. Conversely, overweight participants and those with lower overall CRC knowledge were less likely to accept FIT. In the multivariate model, more frequent physician visits, prior participation in other health screening programs, and previous FIT testing remained independent predictors of higher screening uptake. In contrast, low overall CRC knowledge and poor self-perceived health were independently associated with reduced participation. The detailed results of the univariate and multivariate logistic regression analyses are presented in Table 3. Figures 2A–C illustrates that FIT refusal declined with increasing CRC knowledge scores for symptoms, risk factors, and total knowledge.

**Table 3:** Logistic regression analysis of factors associated with participation in FIT screening.

Variable	Univariate OR (95% CI)	*p*-value	Multivariate OR (95% CI)	*p*-value
Sociodemographic factors				
Sex (Female vs. Male)	1.45 (1.11–1.91)	0.01	1.38 (0.83–2.31)	0.22
Age (Ref: 45–54 years)				
55–64 years	0.83 (0.58–1.17)	0.28	0.66 (0.40–1.09)	0.10
65–74 years	0.80 (0.53–1.23)	0.32	0.72 (0.36–1.44)	0.35
Marital status (Ref: Single)				
Married	0.60 (0.14–2.52)	0.48	–	–
Widowed/Separated	0.96 (0.19–4.80)	0.97	–	–
Education (Ref: Illiterate)				
High school or below	1.51 (0.94–2.44)	0.09	0.68 (0.40–1.15)	0.15
University/Postgraduate	1.20 (0.80–1.80)	0.39	0.63 (0.31–1.29)	0.21
Employment status (Ref: Employed)				
Self-employed	0.67 (0.35–1.29)	0.23	0.90 (0.39–2.06)	0.80
Retired	1.52 (0.94–2.48)	0.09	1.42 (0.77–2.60)	0.26
Unemployed	1.58 (1.09–2.28)	0.02	1.35 (0.73–2.51)	0.34
Residence (Peripheral vs. Main cities)	1.03 (0.76–1.39)	0.86	–	–
Health-related factors				
BMI (Ref: Normal)				
Obese	0.77 (0.51–1.17)	0.23	0.96 (0.53–1.75)	0.90
Overweight	0.52 (0.36–0.76)	<0.001	0.59 (0.35–1.01)	0.05
Smoking status (Ref: Smoker)				
Ex-smoker	0.56 (0.28–1.12)	0.10	–	–
Non-smoker	0.90 (0.49–1.68)	0.75	–	–
Perceived health (Ref: Good)				
Moderate	0.38 (0.28–0.52)	<0.001	0.30 (0.21–0.45)	<0.001
Poor	0.51 (0.15–1.77)	0.29	0.19 (0.05–0.78)	0.02
Physical activity (Ref: Active)				
Moderate	0.79 (0.54–1.16)	0.23	–	–
Sedentary	0.98 (0.66–1.46)	0.91	–	–
Physician visits last 12 months (Ref: 0–1/year)				
2–5 per year	1.24 (0.94–1.63)	0.13	1.57 (1.07–2.31)	0.02
>5 per year	4.60 (1.85–11.44)	0.001	6.07 (1.84–19.99)	0.003
Screening history				
History of other health screenings (Yes vs. No)	2.48 (1.86–3.30)	<0.001	2.02 (1.30–3.14)	0.002
Screening for DM (Yes vs. No)	1.74 (1.32–2.30)	<0.001	–	–
Screening for HTN (Yes vs. No)	1.52 (1.16–1.99)	0.002	–	–
Screening for BC (Yes vs. No)	3.99 (2.20–7.24)	<0.001	–	–
Screening for Osteoporosis (Yes vs. No)	2.03 (1.21–3.38)	0.007	–	–
Previous FIT test (Yes vs. No)	5.91 (3.74–9.35)	<0.001	6.50 (3.61–11.69)	<0.001
CAM knowledge scores (Ref: Good)				
CAM-RF				
Poor	0.84 (0.57–1.24)	0.38	–	–
Intermediate	0.50 (0.33–0.77)	0.002	–	–
CAM-S				
Poor	0.45 (0.29–0.70)	<0.001	–	–
Intermediate	0.37 (0.23–0.60)	<0.001	–	–
CAM-Total				
Poor	0.37 (0.22–0.65)	<0.001	0.34 (0.16–0.73)	0.006
Intermediate	0.27 (0.15–0.48)	<0.001	0.21 (0.09–0.46)	<0.001

OR, Odds ratio; CI, Confidence interval; BMI, Body mass index; DM, Diabetes mellitus; HTN, Hypertension; BC, Breast cancer; FIT, Fecal immunochemical test; CAM, Cancer awareness measure; CAM-S, Symptom knowledge score; CAM-RF, Risk factor knowledge score; CAM-Total, Total knowledge score.

### 3.5 Facilitators to improve colorectal cancer screening uptake among decliners

Among the 240 participants who declined FIT screening, several potential facilitators were identified. The most frequently cited factor was the option to receive the results electronically or by mail (45.8%), followed by encouragement from family members or peers who had previously undergone screening (45.0%). Logistical improvements, including mailing the FIT kit directly to participants (42.5%) and more convenient scheduling of screening and follow-up (42.1%), were also highlighted. Supportive physician–patient communication was considered valuable: 42.5% reported that a dedicated consultation with a primary care provider would increase their willingness to participate, and 42.1% endorsed additional support services to reduce anxiety about the process. Providing step-by-step instructions for obtaining and completing the test was cited by 39.2% of participants as a helpful facilitator.

## 4 Discussion

This study provides the first large-scale FIT-specific assessment of CRC screening in the Qassim region of Saudi Arabia and offers comprehensive insights into both uptake and awareness. The findings showed a high acceptance rate of FIT-based screening, with nearly nine out of 10 eligible participants agreeing to undergo the test. This uptake demonstrates the strong feasibility of scaling up organized programs. In this study, the refusal rate (11.7%) was lower than the rates reported internationally (22, 30), underscoring the feasibility and scalability of implementing organized CRC screening in the region (31). Notably, the demographic and health profiles of the participants were broadly consistent with national patterns, except for a higher proportion of illiterate participants in Qassim (27.2% vs. 3.4% nationally) (32). Despite this, screening acceptance was high, although knowledge scores were low across all educational levels, suggesting that general literacy may not necessarily translate into cancer-specific awareness.

Among those who declined the FIT test, misperceptions were the most prominent barrier. Nearly half believed screening was unnecessary in the absence of symptoms or a family history of cancer, reflecting the confusion between preventive screening and diagnostic evaluation. Psychological concerns, such as fear of results and embarrassment, and logistical challenges, including inconvenience and time constraints, discouraged participation. These findings align with reports from Saudi Arabia (33, 34), the United Arab Emirates (35, 36), and Lebanon (37), all of which identified misperceptions, stigma, and a lack of physician recommendations as common obstacles. Addressing these issues will require culturally tailored education, reassurance from trusted providers, and streamlined and more convenient screening pathways.

A major barrier revealed in this study was the persistently low knowledge of CRC symptoms and risk factors. The mean CAM score was only 8.2 out of 22, with only 15.2% of the participants classified as having good knowledge. Symptom recognition was particularly poor: while blood in stool was identified by half of the respondents, fewer than one-third recognized hallmark signs such as bowel habit changes, abdominal pain, or anemia. Risk factor knowledge was similarly limited, with smoking being the most frequently recognized, but obesity, diet, diabetes, family history, and inflammatory bowel disease were largely under-recognized. These findings are consistent with prior studies in Qatar (38), Lebanon (37), and the UAE (36), as well as with a broader review of the Middle East and North Africa (MENA) region (39). Importantly, participants with poor or intermediate CAM scores were significantly less likely to undergo screening, confirming that awareness is not only a theoretical measure but also a practical determinant of behavior. These findings indicate that substantial CRC knowledge gaps persist and should be addressed through targeted educational interventions integrated into screening delivery.

Despite strong initial acceptance, long-term adherence has emerged as a concern. While most participants accepted FIT screening, fewer than half of those with prior FIT experience adhered to the recommended annual schedule. Only 8.5% correctly identified the yearly interval, and this lack of awareness may partially explain the low regular participation. Inadequate understanding of recommended screening frequency, therefore, represents an important barrier to sustained adherence. Previous studies have similarly demonstrated that inadequate knowledge of screening intervals, together with the absence of reminder systems, are common challenges to maintaining regular participation in FIT-based screening programs (40–42). Sustained effectiveness of the program depends on transforming one-time participation into consistent adherence. Evidence suggests that reinforcing mechanisms such as SMS reminders, electronic health record prompts, and home-delivered FIT kits could improve regular uptake. Simplifying the test instructions and offering step-by-step guidance may also enhance compliance (43, 44).

Multivariate analysis identified key predictors of screening participation. Frequent physician contact and prior engagement in other health screenings were strong facilitators, indicating that individuals embedded in healthcare systems were more likely to comply with CRC screening. In contrast, participants with poorer self-perceived health, limited knowledge, or overweight were less likely to participate. These findings highlight the importance of integrating CRC screening into routine primary care encounters where providers can counsel and motivate patients. Among the decliners, logistical convenience, social encouragement, and dedicated provider consultations were identified as potential facilitators, further reinforcing the value of structural and interpersonal support.

These findings have significant implications. A high initial acceptance suggests that the population is receptive to CRC screening initiatives, but long-term success will depend on embedding annual FIT participation as a community norm. To achieve this, policymakers and healthcare providers must combine targeted education with structural support such as digital reminders, simplified logistics, and stronger provider–patient engagement. By addressing both cognitive and practical barriers, the program can enhance early detection, reduce the disease burden, and improve population-level outcomes.

This study has several strengths, including a large and diverse sample, the use of a validated awareness scale with excellent reliability, and linkage of knowledge levels, sociodemographic characteristics, and health behaviors with screening participation. However, this study has some limitations. Its cross-sectional design prevents drawing causal conclusions, and reliance on self-reported information may have introduced recall or social desirability bias, although the anonymous nature of data collection likely minimized these effects. In addition, the face-to-face, interviewer-administered format may have introduced interviewer bias, as the interviewer’s presence or phrasing could have influenced participants’ responses. To minimize this risk, interviewers received standardized training and used a structured questionnaire. Furthermore, the study was limited to a single region, which may limit the generalizability of the findings to other Saudi Arabian populations. Finally, although barriers and facilitators were explored, the effectiveness of specific interventions was not assessed, leaving this an important area for future research.

Saudi Arabia, with its young median age and steadily increasing life expectancy (45), is undergoing a demographic transition that will expand the population at risk for age-related diseases, including CRC. Given that advancing age is a major determinant of CRC incidence, the country is likely to face a substantial increase in CRC cases in the coming decades. Sustaining and expanding organized screening programs while addressing barriers and knowledge gaps will be essential for reducing future burden.

## 5 Conclusion

In Qassim, FIT screening is widely accepted but poorly maintained, while CRC knowledge is low. Multivariable analysis showed that reduced uptake was independently associated with poorer self-perceived health, fewer physician visits, and low CRC knowledge, whereas prior participation in other health screenings and previous FIT testing predicted higher uptake. Beyond screening behavior, limited knowledge also weakens prevention and delays timely help-seeking. Misconceptions, fear, and logistical challenges further hinder participation, while mailed kits, electronic result reporting, and physician counseling emerged as effective facilitators. For sustainable national control of CRC, efforts must combine accessible, patient-centered delivery with strategies to strengthen the knowledge of symptoms and risk factors, empower individuals to recognize early warning signs, and reinforce regular screening in asymptomatic adults.

## Data Availability

The raw data supporting the conclusions of this article will be made available by the authors, without undue reservation.
