# Supplementary material for: Colorectal cancer awareness and acceptance of fecal immunochemical test screening in Qassim, Saudi Arabia
Source: Front Public Health. 2026 Jan 21;14:1710204. doi: 10.3389/fpubh.2026.1710204 (PMC12868166; doi:10.3389/fpubh.2026.1710204)
Supplement: Supplementary file 1 [file Data_Sheet_1.pdf]

Supplementary Material

Supplementary Appendix 1 – Survey Instrument

Title

Colorectal cancer awareness and acceptance of fecal immunochemical test screening in Qassim, Saudi Arabia

Instructions for Data Collectors (Face-to-Face Administration)

Administer the questionnaire in person. Record responses by marking "√" as chosen by the participant.

Part A. Eligibility Criteria

Instruction: Please answer each question with Yes or No.

#	Question	Response Options
1	Age 45–75 years	□ Yes □ No
2	Meets the national program colorectal screening criteria	□ Yes □ No
3	Willing to participate and provide written consent	□ Yes □ No
4	Capable of completing the survey	□ Yes □ No

Part B. Sociodemographic and Clinical Characteristics

Instruction: Please select the option that best describes you.

#	Question	Response Options
1	Age in years	___ years old.
2	Gender	□ Female □ Male
3	Marital status	□ Single □ Married □ Divorced □ Widowed □ Prefer not to say
4	Education	□ Illiterate □ High school or below □ University and postgraduate □ Prefer not to say
5	Employment status	□ Employed □ Unemployed □ Self-employed □ Retired □ Prefer not to say
6	Residence	□ Central □ Peripheral □ Prefer not to say
7	Height (cm) and weight (kg)	______ cm, ______ kg □ Prefer not to say
8	Smoking status	□ Smoker □ Ex-smoker □ Non-smoker □ Prefer not to say
9	Which of these words best describe your physical health status?	□ Good □ Moderate □ Poor □ Prefer not to say
10	Which of the following best describes your physical activity?	□ Active (≥3 times/week, ≥30 min) □ Moderate (1–3 times/week, ≥30 min) □ Not active (less than once/week)
11	Have you ever participated in the screening of any of the following health conditions?	□ Osteoporosis □ Diabetes mellitus □ Breast cancer □ Other: ______
12	Do you think you are at a higher risk of getting colorectal cancer?	□ Yes □ No □ I don't know
13	Have you done at least one FIT test in the past? (If no, move to Section C)	□ Yes □ No □ I don't know
14	At what age did you take your first FIT test?	___ years old.
15	How many FIT tests did you take in the past?	______ times.
16	Do you follow the regular screening FIT test every year?	□ Yes □ No □ I don't know
17	Time since the last FIT test	□ One year ago □ 1–2 years □ 2–4 years □ >4 years
18	How many times did you visit the doctor's office this year for any reason?	□ 0–1 visit/year □ 2–5 visits/year □ >5 visits/year

Part C. Knowledge of FIT and Screening in Qassim

Instruction: Please answer each question with Yes, No, or I don't know (unless otherwise specified).

#	Question	Response Options
1	FIT test is the standard method of identifying asymptomatic colorectal cancer at an early stage.	□ Yes □ No □ I don't know
2	As far as you can tell, do we have a free screening program in Qassim?	□ Yes □ No □ I don't know
3	At which age should you start taking the FIT test?	□ Above 45 □ Above 55 □ Above 65 □ I don't know
4	How often should you take the FIT test?	□ Once in a lifetime □ Yearly □ Every 5 years □ I don't know

Part D. Knowledge of Colorectal Cancer Symptoms and Risk Factors

Instruction: Please answer each item with Yes, No, or I don't know.

Symptoms (CAM-S)

**Question**	**Response Options**
1. Do you think pain in the back passage could be a sign of bowel cancer?	□ Yes □ No □ I don't know
2. Do you think bleeding from the back passage could be a sign of bowel cancer?	□ Yes □ No □ I don't know
3. Do you think a lump in the abdomen (tummy) could be a sign of bowel cancer?	□ Yes □ No □ I don't know
4. Do you think persistent pain in the abdomen (tummy) could be a sign of bowel cancer?	□ Yes □ No □ I don't know
5. Do you think that tiredness/anemia could be a sign of bowel cancer?	□ Yes □ No □ I don't know
6. Do you think a change in bowel habits (diarrhea, constipation or both) over a period of weeks could be a sign of bowel cancer?	□ Yes □ No □ I don't know
7. Do you think unexplained weight loss could be a sign of bowel cancer?	□ Yes □ No □ I don't know
8. Do you think a feeling that the bowel does not completely empty after using the lavatory could be a sign of bowel cancer?	□ Yes □ No □ I don't know
9. How confident are you that you would notice a bowel cancer symptom?	□ Not at all □ Not very □ Fairly □ Very
10. If you had a symptom that you thought might be a sign of bowel cancer, how soon would you contact your doctor?	□ Immediately □ <1 week □ <1 month □ When free □ Other: ________

Risk Factors (CAM-RF)

**Question**	**Response Options**
11. Do you think bowel disease (e.g., ulcerative colitis, Crohn's disease) increases the risk of bowel cancer?	□ Yes □ No □ I don't know
12. Do you think diabetes increases the risk of bowel cancer?	□ Yes □ No □ I don't know
13. Do you think alcohol consumption increases the risk of bowel cancer?	□ Yes □ No □ I don't know
14. Do you think tobacco smoking increases the risk of bowel cancer?	□ Yes □ No □ I don't know
15. Do you think physical inactivity (<30 min, 5 times/week) increases the risk of bowel cancer?	□ Yes □ No □ I don't know
16. Do you think low fruit and vegetable intake (<5 portions/day) increases the risk of bowel cancer?	□ Yes □ No □ I don't know
17. Do you think eating red or processed meat once a day or more increases the risk of bowel cancer?	□ Yes □ No □ I don't know
18. Do you think a low fiber diet increases the risk of bowel cancer?	□ Yes □ No □ I don't know
19. Do you think being overweight (BMI >25) increases the risk of bowel cancer?	□ Yes □ No □ I don't know
20. Do you think being over 70 years old increases the risk of bowel cancer?	□ Yes □ No □ I don't know
21. Do you think having a close relative with bowel cancer increases the risk of bowel cancer?	□ Yes □ No □ I don't know
22. In the next year, who is most likely to develop bowel cancer?	□ 20 y/o □ 40 y/o □ 60 y/o □ Not age-related

Part E. Perceived Barriers to FIT

Instruction: Please indicate whether you Agree, Disagree, or are Unsure for each statement.

**Barrier**	**Response Options**
1. Not at risk due to absence of symptoms	□ Agree □ Disagree □ Unsure
2. Not at risk due to healthy lifestyle	□ Agree □ Disagree □ Unsure
3. Not at risk due to absence of family history	□ Agree □ Disagree □ Unsure
4. Lack of time	□ Agree □ Disagree □ Unsure
5. Fear of positive result	□ Agree □ Disagree □ Unsure
6. Embarrassment	□ Agree □ Disagree □ Unsure
7. Inconvenience of the test	□ Agree □ Disagree □ Unsure
8. Doubt about effectiveness	□ Agree □ Disagree □ Unsure
9. Cannot return sample	□ Agree □ Disagree □ Unsure
10. Hesitant about colonoscopy after a positive result	□ Agree □ Disagree □ Unsure

Open-ended response: Other (specify) _____________________________

Part F. Behavioral Facilitators of Screening Uptake

Instruction: Please indicate whether you Agree, are Neutral, or Disagree with each statement.

Facilitator	Response Options
1. Ability to receive results electronically/by mail	□ Agree □ Neutral □ Disagree
2. Easier booking of FIT/colonoscopy appointments	□ Agree □ Neutral □ Disagree
3. Explanation of benefits by healthcare providers	□ Agree □ Neutral □ Disagree
4. Support services to address concerns/fears	□ Agree □ Neutral □ Disagree
5. Encouragement from family/peers	□ Agree □ Neutral □ Disagree
6. Information on test steps and follow-up	□ Agree □ Neutral □ Disagree
7. Receiving/returning FIT kit by mail	□ Agree □ Neutral □ Disagree

Open-ended response: Other (specify) _____________________________
